# Phenolics, Flavonoids, Antioxidant Activity and Cyanogenic Glycosides of Organic and Mineral-base Fertilized Cassava Tubers

**DOI:** 10.3390/molecules17032378

**Published:** 2012-02-27

**Authors:** Nur Faezah Omar, Siti Aishah Hassan, Umi Kalsom Yusoff, Nur Ashikin Psyquay Abdullah, Puteri Edaroyati Megat Wahab, Uma Rani Sinniah

**Affiliations:** 1 Department of Crop Science, Universiti Putra Malaysia, Serdang 43400, Selangor, Malaysia; Email: nurfaezahomar@gmail.com (N.F.O.); nurashikin@putra.upm.edu.my (N.A.P.A.); putri@agri.upm.edu.my (P.E.M.W.); umarani@agri.upm.edu.my (U.S.); 2 Department of Biological Science, Universiti Putra Malaysia, Serdang 43400, Selangor, Malaysia; Email: umikay@science.upm.edu.my

**Keywords:** *Manihot esculenta*, cassava tuber quality, fertilizer sources, phytochemical compounds, antioxidant activity

## Abstract

A field study was conducted to determine the effect of organic and mineral-based fertilizers on phytochemical contents in the tubers of two cassava varieties. Treatments were arranged in a split plot design with three replicates. The main plot was fertilizer source (vermicompost, empty fruit bunch compost and inorganic fertilizer) and sub-plot was cassava variety (Medan and Sri Pontian). The amount of fertilizer applied was based on 180 kg K_2_O ha^−1^. The tubers were harvested and analyzed for total flavonoids, total phenolics, antioxidant activity and cyanogenic glucoside content. Total phenolic and flavonoid compounds were determined using the Folin-Ciocalteu assay and aluminium chloride colorimetric method, respectively. Different sources of fertilizer, varieties and their interactions were found to have a significant effect on phytochemical content. The phenolic and flavonoid content were significantly higher (*p* < 0.01) in the vermicompost treatment compared to mineral fertilizer and EFB compost. The total flavonoids and phenolics content of vermicompost treated plants were 39% and 38% higher, respectively, than those chemically fertilized. The antioxidant activity determined using the DPPH and FRAP assays were high with application of organic fertilizer. Cyanogenic glycoside levels were decreased with the application of organic fertilizer. Among the two types of compost, vermicompost resulted in higher nutritional value of cassava tubers. Medan variety with application of vermicompost showed the most promising nutritional quality. Since the nutritional quality of cassava can be improved by organic fertilization, organic fertilizer should be used in place of chemical fertilizer for environmentally sustainable production of better quality cassava.

## 1. Introduction

Cassava (*Manihot esculenta* Crantz) has the ability to grow on the poorest soils and left to compete with weeds. This ability has led many to think that cassava, either when grown alone or as an intercrop does not require high soil fertility and does not respond to fertilizer application. Farmers do not fertilize cassava because they are assumed to be content with the minimal yields obtained from using limited inputs or even from their infertile soils. The indifference towards low productivity can be attributed to the low and unstable prices of cassava tubers. However, fertilizer requirements for optimum yield in cassava is determined by the soil fertility status of the farmland, cropping system, and the rainfall pattern during the growing season [1]. Cassava had been reported to respond to good soil fertility and adequate fertilizer [2]. It commonly requires some application of N and K fertilizers for maximum growth and yields [2,3], and lack of K affects its response to N and P. 

Many factors influence the phyto-nutritional status of crops. Genotypic differences are the main factor in causing a large variation in vitamin content [4], antioxidant capacity and phenolic content [5]. According to Bok *et al*. [6], different varieties will give different levels of antioxidant compounds. Other factors include climatic conditions and cultural practices [7]. Fertilization has been reported to have an influence on the phyto-nutritional quality of crops. Inorganic fertilizer is said reduce the antioxidant levels while organic fertilizer was proven to enhance the antioxidant content in plants [8]. Applying fertilizer, particularly in inorganic form, in excess of plant requirements can increase the chances of fertilizer loss and environmental pollution. 

In the past, agricultural production was focused on maximizing the quantity of crop produced for commercial markets, hence in common agricultural practices, compound fertilizer has been used on field grown cassava. Nowadays however, health conscious consumers are interested in optimizing the nutritional composition with minimal chemical residues on foods produced through environmentally friendly agricultural practices. Substituting chemicals with organic fertilizers is one of the common principles in this production system. Compost and vermicompost have been widely applied as sources of nutrients. 

The nutritional quality of organically and conventionally grown plants has been compared mainly in terms of macronutrients, vitamins, and minerals. Organically produced vegetables have higher levels of vitamin C, iron, magnesium and phosphorus and less nitrates and lower amounts of some heavy metal [9]. There are very few studies that have compared the levels of antioxidant compounds in organically and conventionally grown products. Asami *et al*. [10] reported that there were significantly higher total phenolics in organically grown marrionberries (620 mg/100 g fresh weight) as compared to those treated with a conventional fertilization method (412 mg/100 g), whereas Perez-Lopez *et al*. [11] reported that organic farming had a significant effect on nutritional content in peppers, increasing the vitamin C activity, total phenolic compound and carotenoid contents. The total antioxidant capacity in head cabbage was also significantly increased with organic fertilizers [12]. Despite the fact cassava is a staple food for 500 million people and grown in more than 90 countries [13], there is no information currently available on the effect of fertilizer sources in relation to the phytochemical compounds in cassava. The natural dietary antioxidant content in food has been the focus of much investigation in recent years due to its contribution in protection against diseases. Therefore, the major objective of this study was to determine the effect of inorganic and various organic fertilizer sources on antioxidant activity in two cassava varieties. 

## 2. Results and Discussion

**Figure 1:**
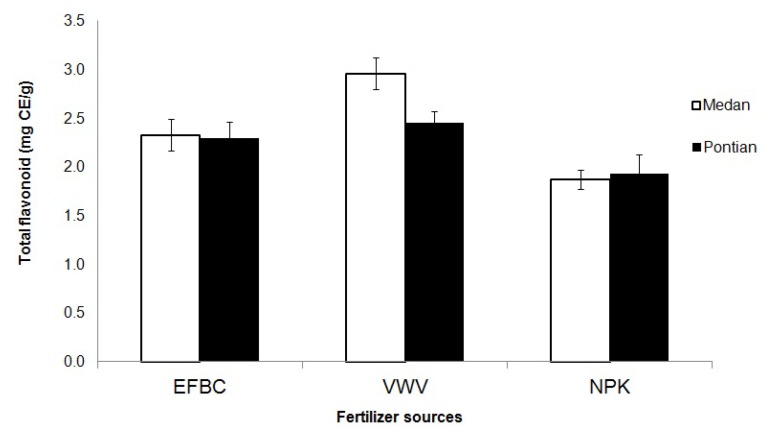
Flavonoids content in tubers of cassava treated with different fertilizer sources.

Phenolic compounds are great of importance in terms of the nutritional and commercial properties of agricultural products, through their contribution to sensory properties such as colour and flavor [11,14]. The total phenolic content was measured in terms of gallic acid equivalent using the Folin Ciocalteu reagent (standard curve equation: y = 1.961 − 0.001x, R^2^ = 0.553). The effects of fertilizers and cassava varieties on total phenolic content and total flavonoid are shown in Table 1. Total phenolic and flavonoid content were found to be significantly higher after organic treatments compared to inorganic fertilizer (Table 2). The highest total phenolic content was observed using vermicompost (10.88 mg GAE/g fw) compared to inorganic fertilizer (8.35 mg GAE/g fw). Differences in flavonoid content of Medan and Pontian varieties was only found with vermicompost (Figure 1).

**Table 1:** ANOVA of Means Square [MS(Pr > F)] for phytochemical compound in tuber.

Source	Total phenolics	Total flavonoids	DPPH scavenging assay	FRAP scavenging assay	Cyanogenic glycoside
Fertilizer (F)	259.23 *	53.76 **	502.98 **	672.65 **	0.022 *
Variety (V)	134.48 *	5.44 *	494.02 **	0.375ns	0.002ns
F × V	56.91 *	17.43 **	45.34 **	13.31 *	0.001ns
CV (%)	2.91	2.75	3.68	3.55	9.02

ns, **, * non significant or significant at *p* ≤ 0.01, *p* ≤ 0.05, respectively.

**Table 2:** Phytochemical compounds in tuber of cassava varieties as affected by fertilizer sources.

Source	Total phenolics (mg GAE/g)	Total flavonoids (mg CE/g)	DPPH scavenging assay (%)	FRAP scavenging assay (%)	Cyanogenic glycoside (mg/100 g)
Fertilizer source VWV	10.88a ^z^	2.71a	67.30a	68.11a	0.40b
EFBC	9.44b	2.32b	54.70b	54.45b	0.42b
Inorganic	8.35c	2.18c	44.37c	50.08c	0.51a
Variety Medan	9.83a	2.47a	47.58a	37.50a	0.43
Sri Pontian	9.20b	2.30b	37.11b	37.21a	0.45

^z^ Means with same letter are not significantly different by LSD, at 5%.

The significant positive correlation shown in Table 3 (r = 0.62) between total phenolic and flavonoid compounds indicates that an increase in phenolics was followed by an increase in total flavonoids. Both were found to be highly correlated with antioxidant activity. Since organically fertilized plants had high phenolics and flavonoids, higher DPPH scavenging activity and FRAP scavenging activity were observed. Fertilization has been reported to have influence on the phyto-nutritional quality of crops. Inorganic fertilizer is said to reduce the antioxidants while organic fertilizer was proven to enhance the antioxidant content in plants [8]. In addition, organic fertilizers increased the content of ascorbic acid and total phenolics in tomato [15]. High yield and quality of broccoli was also been reported by using organic fertilizers [16]. In the case of cassava, our result indicated that sources of fertilizer had a significant influence on the level of phytochemical compounds in field grown cassava.

**Table 3:** Correlation coefficients between total phenolic compounds, total flavonoid content and antioxidant activity determination assays (DPPH and FRAP) of cassava.

	TPC	TFC	DPPH	FRAP
TPC	-	0.62 *	0.83 **	0.82 **
TFC	-	-	0.61ns	0.74 **
DPPH	-	-	-	0.79 **
FRAP	-	-	-	-

For correlation coefficients, n = 18; ns, *, ** Non significant or significant at *p* ≤ 0.05 and *p* ≤ 0.01, respectively.

**Figure 2:**
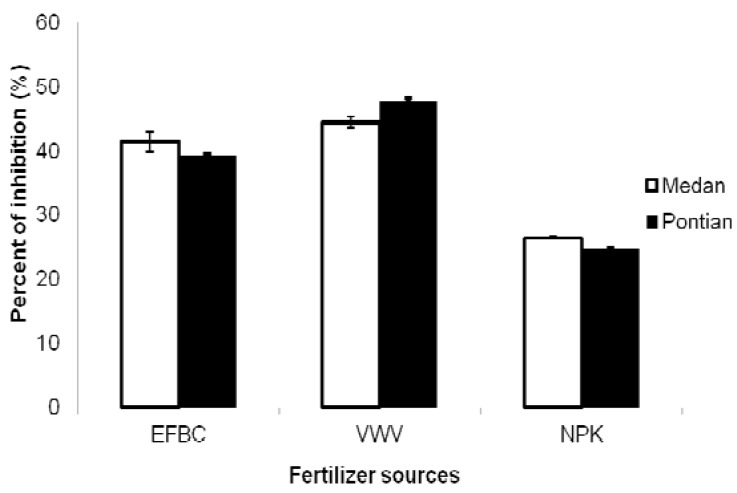
DPPH scavenging activities of cassava varieties treated with different fertilizer sources.

The antioxidant activity of plant extracts were assessed by the DPPH free radical scavenging method. This is a stable free radical whose color changes from violet to yellow when is reduced by hydrogen donation. The DPPH method has been widely applied for estimating antioxidant activity in recent years [17]. It was observed that the percentage of inhibition was found to be dependent on the source of fertilizer and the cassava varieties (Figure 2). 

The highest value of DPPH scavenging activity (67.30%) was observed from vermicompost treatment (Table 2). 

Based on the antioxidants activity, the percentage of inhibition was increased by using vermicompost as fertilizer source. Among the two varieties, the highest percent based on DPPH inhibition was found in the Medan variety (47.58%). There was a significant positive correlation (*p* ≤ 0.01) between TPC and the DPPH assay, (r = 0.83) (Table 3). These results showed that cassava possesses strong antioxidant activity and can be used as a good source of natural antioxidants.

**Figure 3:**
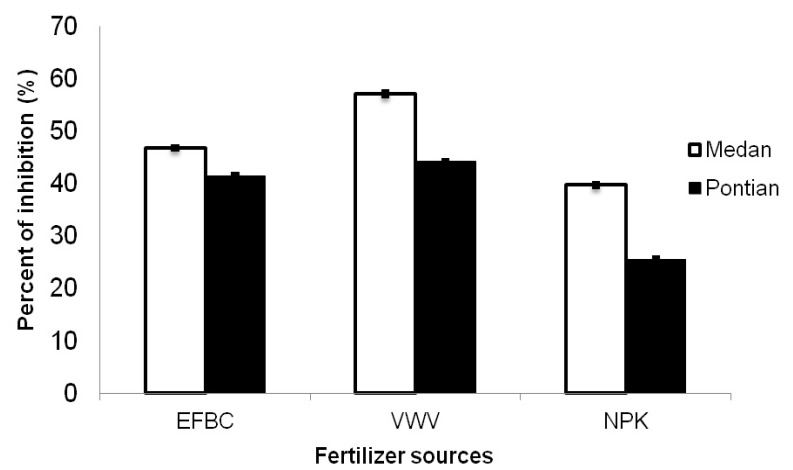
FRAP scavenging activities of cassava varieties treated with different fertilizer sources.

The FRAP assay is widely used in the evaluation of antioxidant components in dietary polyphenols [18]. When samples react with FRAP solution, a dark blue color will appear which corresponds to the ferrous tripyridyltriazine complex. The extracts which exhibit antioxidant activity such as Medan treated with vermicompost produced more ferrous tripyridyltriazine complexes compared to Pontian. Ferrous tripyridyltriazine complexes were produced as product from the reaction in which the samples had the ability to reduce Fe^3+^ to Fe^2+^. The greater amount of Fe^3+^ reduced to Fe^2+^, the higher the total antioxidant content observed. In all treatments, the radical scavenging activity of extracts increased with increasing concentrations of the plant extracts [19]. Similarly, the percentage of FRAP inhibition was found to be dependent on the source of fertilizer and the cassava varieties (Figure 3). 

Among the three nutrient sources applied, the plant with the vermicompost application had significantly greater antioxidant activities, followed by EFB compost and inorganic fertilizer. Medan and Pontian showed no significant differences in FRAP scavenging activity (Table 2). FRAP assay showed a significant (*p* ≤ 0.01) positive correlation (r = 0.74), with total phenolic compounds (Table 3). Several researches on the phenolic constituents and antioxidant activities in various plants have been conducted. According to [11], organic farming had a positive effect on the nutritional quality content of sweet peppers (*C. annuum* cv. Almuden), increasing the vitamin C activity and the level of phenolic compounds. The different influences of compost and vermicompost on physiological activities could be due to fundamental differences between the composting and vermicomposting processes. In addition, enzymatic activity of worms in vermicompost as well as the presence of beneficial microorganism that maybe affected the physiological activities [20].

The raw roots of cassava plant can be toxic due to the presence of natural nitrile (-CN) compounds classed as cyanogenic glycosides [21]. The leaves, roots and stems of cassava contain potentially toxic levels of cyanogenic glycosides [linamarin (95%) and lotaustralin (5%)] [22] as a defence mechanism against attack by predators. The maximum recommended cyanide levels in foods established by the Codex Alimentarius Commission of the FAO/WHO is 10 mg CN equivalents/kg dry weight. Although in general the level of cyanogenic glycosides found in this experiment was lower than the FAO maximum recommended level, organic fertilizer application resulted in significantly lower cyanide content compared to inorganic fertilizer (Table 2). The results showed no significant difference was observed between both varieties. There was no interaction effect (FxV) observed between fertilizer and variety on cyanogenic glycosides (Table 1). The low content of these anti-nutrients using organic fertilizer would therefore permit the absorption of these elements which they form complexes with. Anti-nutrients are natural or synthetic compounds that interfere with the absorption of nutrients. These cyanogens are distributed widely throughout the plant, with large amounts in the leaves and the root cortex (skin layer), and generally smaller amounts in the root parenchyma (interior). However, some of these toxic substances can be reduced during processing of cassava, which include cooking, fermentation and soaking [23].

## 3. Experimental

### 3.1. General

The factorial experiment was conducted on sandy clay loam soil with the pH of 5.7 under open field condition from January 2010 to October 2010. The treatments were arranged in split plot design with three replications. The main plot was fertilizer sources consisting of vegetable waste vermicompost (N: 2.32%; P: 1.54%; K: 1.06%) empty fruit bunch compost (N: 1.46%; P: 1.47%; K: 2.58%) and inorganic fertilizer (N: 15%; P: 15%; K: 15%). The sub plot was cassava Medan and Sri Pontian varieties. Stem cuttings (20–25 cm in length) were planted at 1 m × 1 m. There were 2 m gaps between treatments and 1 m alleys between plots. The amount of fertilizer applied was calculated based on 180 kg·h^−1^ of K_2_O. The soil was thoroughly plowed and mixed with organic compost during planting while inorganic fertilizer was applied equally at two and twenty weeks after planting. The soil was covered with organic mulch and sprinkler irrigated. The tubers were harvested after nine months and analyzed for total phenolic acids and total flavonoids content, cyanogenic glycosides content and DPPH free radical scavenging and FRAP scavenging assays. The data were analyzed using analysis of variance and significant differences between means was done by least significant difference test (*p* < 0.05).

### 3.2. Extraction of Total Phenolic Acids and Total Flavonoids

Extraction of total phenolic acid and total flavonoid assay was conducted using the modified method of [24]. Cassava tuber (0.5 g) was ground using a mortar and pestle. These samples were homogenized with distilled water (50 mL) and transferred to a covered flask. Then the mixture was centrifuged for 5 min at 14,000 rpm. The supernatant was collected and used for total phenolic acids and total flavonoids quantification.

### 3.3. Total Phenolics

Total phenolic (TP) compounds were assayed as described by [24] using the Folin-Ciocalteu assay. Extract (1 mL) was added to a flask containing distilled water (9 mL). Then Folin-Ciocalteu’s phenol reagent (1 mL) was added and the mixture was mixed thoroughly. After 5 min, 7% sodium carbonate (10 mL) was added. The mixture was diluted to 25 mL with the addition of distilled water (4 mL) and allowed to stand at room temperature for 90 min. The absorbance was monitored using a spectrophotometer (U-2001, Hitachi Instruments Inc., Tokyo, Japan) at 750 nm. TP content was expressed as mg gallic acid equivalents (GAE)/g samples.

### 3.4. Total Flavonoid Assay

Total flavonoid was determined according to [24] using the aluminum chloride colorimetric method. Extract (1 mL) was added to distilled water (4 mL) in a flask. Then, 5% NaNO_2_ (0.3 mL) was added. After 5 min, 10% AlCl_3_ (0.3 mL) was added and after 6 min, 1 M NaOH (2 mL) was added. The mixture was diluted to 10 mL with distilled water. The absorbance of the solution was measured at 510 nm using a spectrophotometer (U-2001, Hitachi Instruments Inc., Tokyo, Japan). The results were expressed as mg catechin equivalents (CE)/g samples. 

### 3.5. Extraction of Antioxidant Compounds

Extraction of antioxidant compounds was conducted employing the method modified by [25]. Tuber (0.5 g) was cut into small pieces at placed in a 150 mL conical flask. A total volume of 25 mL of distilled water was added and covered with aluminum foil. The conical flasks containing the samples were placed in orbital shaker for 1 h in the dark at room temperature. Then the samples were filtered using Whatman No. 1 paper. The extracts were stored at 0–4 °C before analysis.

### 3.6. DPPH Free Radical Scavenging Assay

1,1-Diphenyl-2-picryl-hydrazyl (DDPH) was purchased from Sigma-Aldrich (St. Louis, MO, USA). The DPPH free radical test was conducted using the method of [25]. The initial absorbance of DPPH in methanol was measured at 515 nm until the absorbance remained constant. Extracts (40 µL) were added to alcohol solution of DPPH (3 mL, 0.1 mM). The samples were first kept in a dark place at room temperature and after 30 min the absorbance was measured using a spectrophotometer (U-2001, Hitachi Instruments Inc., Tokyo, Japan) at 515nm. The percent of inhibition was determined using the formula: percent of inhibition (%) = [(A515 of control − A515 of sample)/A515 of control] × 100. 

### 3.7. Ferric Reducing Antioxidant Power Assay (FRAP)

The determination of the total antioxidant activity in the extract using the FRAP assay followed after a modified method reported by [25]. Extract (200 µL) was added to FRAP reagent [3 mL, 10 parts 300 mM sodium acetate buffer at pH 3.6, 10 mM 2,4,6-tri(2-pyridyl)-*s*-triazine (TPTZ) solution and 20 mM FeCl·6H_2_O solution) and the reaction mixture was incubated in a water bath at 37 °C for 30 min. The increase in absorbance was measured at 593 nm using a spectrophotometer (U-2001, Hitachi Instruments Inc., Tokyo, Japan). The percent of antioxidant was calculated using the formula: percent of antioxidant (%) = [(A593 of sample − A593 of control)/A593 of sample] × 100. 

### 3.8. Determination of Cyanogenic Glycoside

The alkaline picrate method of [21] was used to determine cyanogenic glycoside in cassava. Roots (5.0 g) were weighed and dissolved in distilled water (50 mL) in conical flasks. The mixtures were allowed to stay overnight and filtered. The filtrates were collected and alkaline picrate solution (4 mL) was added to each and incubated in water bath for 15 min. When the mixture turned to reddish brown the absorbance was taken at 490 nm using a spectrophotometer (U-2001, Hitachi Instruments Inc., Tokyo, Japan). Different concentration of hydrogen cyanide (HCN) was prepared containing 0.02 to 0.10 mg/mL cyanide for the blank mixtures. The measurement of cyanogenic glycoside was repeated in triplicate. The cyanide content was extrapolated from the cyanide curve. 

## 4. Conclusions

In summary, it can be concluded that nutrient sources can have significant effects on antioxidant activity and phenolic metabolites in cassava. Organic fertilizer should be used in place of chemical fertilizer for environmentally sustainable production of better quality cassava. The results indicated that application of vermicompost and empty fruit bunch compost can enhance the antioxidant activities of field grown cassava. Application of inorganic fertilizer increased the level of cyanide in cassava. Medan variety with application of vermicompost showed the most promising nutritional quality. Phytochemical composition was significantly improved with the application of organic fertilizer and vermicompost showed better effects than EFB compost.
